# Intake of 3 Eggs per Day When Compared to a Choline Bitartrate Supplement, Downregulates Cholesterol Synthesis without Changing the LDL/HDL Ratio

**DOI:** 10.3390/nu10020258

**Published:** 2018-02-24

**Authors:** Bruno S Lemos, Isabel Medina-Vera, Christopher N Blesso, Maria Luz Fernandez

**Affiliations:** 1 Department of Nutritional Sciences, University of Connecticut, Storrs, Mansfield, CT 06269, USA; bruno.lemos@uconn.edu (B.S.L.); christopher.blesso@uconn.edu (C.N.B.); 2 Departamento de Metodologia de Investigacion, Instituto Nacional de Pediatria, CD Mexico 04530, Mexico; isabelj.medinav@gmail.com

**Keywords:** dietary cholesterol, eggs, cholesterol metabolism, apolipoproteins, choline bitartrate, cardiovascular disease, gene expression

## Abstract

Cardiovascular disease (CVD) risk is associated with high concentrations of low-density lipoprotein cholesterol (LDL-C). The impact of dietary cholesterol on plasma lipid concentrations still remains a concern. The effects of egg intake in comparison to choline bitartrate supplement was studied in a young, healthy population. Thirty participants were enrolled for a 13-week intervention. After a 2-week run-in period, subjects were randomized to consume either 3 eggs/day or a choline bitartrate supplement (~400 mg choline for both treatments) for 4-weeks each. After a 3-week washout period, they were allocated to the alternate treatment. Dietary records, plasma lipids, apolipoproteins (apo) concentrations, and peripheral blood mononuclear cell expression of regulatory genes for cholesterol homeostasis were assessed at the end of each intervention. Dietary intakes of saturated and monounsaturated fat were higher with the consumption of eggs compared to the choline period. In addition, higher plasma concentrations of total cholesterol (7.5%), high density lipoprotein cholesterol (HDL-C) (5%) and LDL-C (8.1%) were observed with egg consumption (*p* < 0.01), while no change was seen in LDL-C/HDL-C ratio, a key marker of heart disease risk. Compared to choline supplementation, intake of eggs resulted in higher concentrations of plasma apoA-I (8%) and apoE (17%) with no changes in apoB. Sterol regulatory element-binding protein 2 and 3-hydroxy-3-methylglutaryl-CoA reductase expression were lower with egg consumption by 18% and 31%, respectively (*p* < 0.05), suggesting a compensation to the increased dietary cholesterol load. Therefore, dietary cholesterol from eggs appears to regulate endogenous synthesis of cholesterol in such a way that the LDL-C/HDL-C ratio is maintained.

## 1. Introduction

Consumption of foods high in fat and cholesterol have been of major concern due to the increasing prevalence of cardiovascular disease (CVD) worldwide [1]. High concentrations of plasma low-density lipoprotein cholesterol (LDL-C) have been shown to be associated with an increased risk for CVD [2]. In contrast, studies have shown that an increase of 1 mg/dL in high-density lipoprotein cholesterol (HDL-C) is related to 2% and 3% reductions in CVD risk in men and women [3]. Therefore, lifestyle interventions typically focus on decreasing plasma LDL-C and, in some cases, increasing HDL-C concentrations. Therefore, the LDL-C/HDL-C ratio has become a key biomarker for CVD risk. Research targeting the pathogenesis, development, and causes of CVD in various populations is of great interest. The accumulation of lipid in the arterial wall during atherosclerosis can prompt a cascade of events that may result in heart attack, stroke, or death [4]. This occurs when LDL particles are modified [5] and consequently enter the intima where they are engulfed by macrophages resulting in foam cell formation [6]. The uptake of oxidized LDL is mainly mediated by scavenger receptors expressed on the surface of macrophages [7]. Eventually, foam cells within the arterial intima will accumulate and form a fatty streak. This will then result in a fibrous cap, which may ultimately rupture to cause a thrombus [8], triggering cardiovascular events. In regards to cholesterol regulation, cholesterol carried by LDL has been postulated to regulate endogenous cholesterol biosynthesis through feedback mechanisms in order to maintain baseline circulating blood cholesterol concentrations [9].

While LDL particles are known to promote CVD, HDL particles have many functionalities that may reduce CVD-related events, through the prevention of lipoprotein modification and maintenance of cholesterol homeostasis [10]. For example, HDL is responsible for reverse cholesterol transport from extrahepatic tissues to the liver in order to clear cholesterol from the body, primarily by utilizing cholesterol as a precursor for bile acid synthesis [11]. Another important property of HDL particles is their antioxidant function in regards to carrying fat-soluble vitamins, such as vitamin E, as well as the carotenoids, lutein, and zeaxanthin [12]. 

Eggs have been investigated as a food associated with CVD risk, due to the cholesterol content in egg yolk [13]. The 2015–2020 Dietary Guidelines for Americans (DGA) removed the longstanding 300 mg/day intake limit for dietary cholesterol from their recommendations, citing the need for further research examining the relationship between dietary cholesterol and blood cholesterol [14]. Recent studies have shown that consumption of eggs in a healthy population improved HDL functionality, increased plasma carotenoids and maintained LDL-C/HDL-C ratio [15,16,17]. Additionally, egg intake for 12 weeks had the same effects in a population with metabolic syndrome [18,19]. However, eggs may also be seen as an unhealthy food because of their high content of choline [13]. In recent years, studies have shown a relationship between dietary choline and atherosclerosis progression, due to choline being a precursor of a metabolite known as trimethylamine *N*-oxide (TMAO) [20]. Studies conducted in rodents on the biological activity of TMAO indicated that it increases the expression of scavenger receptors responsible for the uptake of modified LDL, which would enhance the formation of foam cells during atherosclerosis [21]. We have demonstrated that fasting plasma TMAO concentrations do not increase with the consumption of 3 eggs in healthy individuals, while increasing plasma choline [17]. 

The objective of this study was to test the effects of consuming 3 eggs per day versus the equivalent amount of supplemental choline on lipid metabolism-related biomarkers of CVD. We hypothesized that the additional dietary cholesterol from eggs would downregulate expression of markers in cholesterol biosynthesis pathway, impacting plasma cholesterol and apolipoprotein (apo) concentrations. 

## 2. Materials and Methods

### 2.1. Subjects and Experimental Design

Thirty healthy men and women were recruited for a 13-week crossover intervention. The number of subjects was supported by a previous study where 3 eggs was sufficient to detect a difference in plasma choline and plasma cholesterol [17] with a *Z* value of 1.96 (95% confidence interval). Therefore, enrolling 25 subjects was an estimate to observe difference among treatments. For this reason, a total of 30 subjects were recruited to compensate for attrition. Primary inclusion criteria for the study were: age 18–30 years, body mass index (BMI) 18.5–29.9 kg/m^2^, blood pressure (BP) within normal values, healthy lipid profile, and willingness to consume 3 eggs daily and choline supplement for 4 weeks each. Exclusion criteria consisted of previous diagnoses of liver disease, renal disease, diabetes, cancer, history of stroke, or heart disease. Additionally, intake of glucose-lowering medication or supplements, allergy to eggs or components of choline supplement, vegan or vegetarian, or antibiotic use in the previous month were exclusion criteria. Other anthropometric and plasma parameter exclusions were: BP ≥ 140/90 mmHg (average of three readings), total cholesterol ≥ 240 mg/dL, plasma triglycerides ≥ 500 mg/dL, plasma glucose ≥ 126 mg/dL, plasma creatinine ≤ 0.5 or ≥0.9 mg/dL for females and ≤0.7 or ≥1.2 mg/dL for males.

The University of Connecticut Institutional Review Board approved the protocol (#H16-194), and all participants signed the consent forms prior to screening. This clinical trial was registered at clinicaltrials.gov (Protocol #NTC03142763).

After screening and qualification, participants began a 2-week run-in period where consumption of any eggs or egg-based foods was prohibited. Abstinence from consuming foods high in choline was required throughout the whole intervention according to a list provided by researchers. Following the run-in period, subjects were randomized to the interventions, egg or choline supplement group. Next, participants consumed 3 eggs/day as their first meal for 4 weeks (EGGS), or took 1 ½ tablets of choline bitartrate supplement with breakfast/first meal for 4 weeks (CHOLINE). Subsequently, subjects went through a washout period of 3 weeks, and then started the alternate intervention. Participants were instructed to consume only the eggs provided by researchers during the egg arm, while during the choline intervention no egg or egg-based foods were allowed. All parameters below were measured at the end of each treatment. Large, grade A, white eggs were obtained from a local supermarket (Big Y, Tolland, CT, USA), Eggs contained approximately 185 mg of cholesterol. Choline bitartrate supplement was obtained from Best Naturals (Kenilworth, NJ, USA) and each tablet contained 265 mg of choline. In order to make equivalent to the amount of choline in three eggs (~390 mg), participants had to consume 1 ½ tablets (~397.5 mg). No specific instructions were given regarding egg preparation. Lastly, subjects were required to maintain their diet and lifestyle throughout the intervention.

### 2.2. Dietary Records

Assessment of diet was conducted through the analysis of 3-day diet records completed by participants during each arm. Participants were given instructions on how to fill out the dietary records, and a reminder to maintain the same diet throughout the study was provided at each visit. Nutrition Data Systems for Research software (2016), developed by the Nutrition Coordinating Center, University of Minnesota, Minneapolis, MN, USA, was used to analyze the dietary records.

### 2.3. Anthropometrics and Plasma Parameters

An electronic scale was used to measure weight, and it was recorded to the nearest 0.1 kg. Height was measured on a stadiometer to the nearest 0.5 cm. Body mass index (BMI) was calculated by dividing weight in kg by the square of height in meters (kg/m^2^). Blood pressure was measured with a portable automatic blood pressure cuff (Omron HEM 7320-Z, Bolingbrook, IL, USA), and participants were asked to sit quietly for 5 min prior to measurement, to obtain an average of 3 readings. Before the end of each intervention arm, participants were asked to fast for 12 h, and 70 mL of blood was collected in ethilendiaminetetraacetic acid-coated vacutainer tubes. 

### 2.4. Plasma Lipids, Plasma Glucose, and Creatinine

About 30 mL of blood was centrifuged to separate plasma for biochemical parameters analyses. Using an automated spectrophotometer (Cobas c-111, Roche Diagnostics, Indianapolis, IN, USA) plasma glucose, triglycerides, total cholesterol, HDL cholesterol (HDL-C), and creatinine were measured. To calculate LDL-C, the Friedewald Equation was used [22]. LDL-C/HDL-C ratio was also calculated. Estimated Glomerular Filtration Rate (eGFR) was calculated using the Modification of Diet in Renal Disease (MDRD) formula [23].

### 2.5. Apolipoproteins Analysis

Quantification of plasma apoA-I, apoB, and apoE was done simultaneously with a commercially available multiplex kit (Invitrogen, Waltham MA, USA) and a Luminex MAGPIX instrument (Luminex Corporation, Austin, TX, USA). 

### 2.6. Peripheral Blood Mononuclear Cell Isolation

The remaining 40 mL of fasting blood was kept on ice to isolate peripheral blood mononuclear cells (PBMCs) by density gradient centrifugation, using Ficoll-Paque PREMIUM (GE Healthcare, Uppsala, Sweden) based on the manufacturer’s instructions. In brief, whole blood was diluted with sterile phosphate buffered saline, layered over Ficoll-Paque PREMIUM, and then centrifuged at 400× *g* for 35 min to separate the buffy coat containing the PBMCs. Next, the buffy coat was separated, washed twice with phosphate buffered saline, and resuspended in fetal bovine serum. Freshly isolated PBMCs were used for RNA isolation. 

### 2.7. Quantitative Real-Time Polymerase Chain Reaction

Gene expression was assessed by mRNA expression in PBMCs using quantitative real-time polymerase chain reaction (qRT-PCR). Using IBI Isolate reagent (IBI Scientific, Peosta, IA, USA) RNA was isolated from fresh PBMCs. Following RNA extraction, 1 µg of RNA was treated with DNase I (Thermo Scientific, Waltham, MA, USA) and reverse-transcribed by iScript transcriptase kit (Bio-Rad, Hercules, CA, USA) using a Bio-Rad C1000 Thermal Cycler (Bio-Rad, Hercules, CA, USA). SYBR Green was used for qRT-PCR analysis with a Bio-Rad CFX96 system (Bio-Rad, Hercules, CA, USA). Primer sequences were designed according to the GenBank database for the genes of interest: 3-hydroxyl-3-methyl-glutaryl-coenzyme A reductase (HMGCR), low-density lipoprotein receptor (LDLR), sterol regulatory element-binding protein 2 (SREBP2), shown in Table 1. Expression of mRNA values was calculated using the threshold cycle (Ct) value. Relative expression levels of each target gene were calculated using the comparative 2^−∆∆*C*T^ method following normalization to glyceraldehyde 3-phosphate dehydrogenase (GAPDH) mRNA expression [24].

### 2.8. Statistical Analysis

SPSS version 25 (IBM Corp., Chicago, IL, USA) was used for all statistical analyses. Paired Student’s *t* test was used to calculate the difference between interventions, EGGS vs CHOLINE. Grubb’s test was used for outliers as noted in results. Level of significance for all results was set at *p <* 0.05. All data are reported as mean ± SD.

## 3. Results

For this intervention, a total of thirty participants were enrolled in January 2017, but only twenty-nine completed the study. The discontinuing of one participant was due to personal reasons that affected compliance. Baseline data are shown in Table 2 separated by gender. All participants were considered healthy based on their BMI, blood pressure, lipid profile, plasma fasting glucose, creatinine, and eGFR. None of the participants were taking cholesterol lowering medications, and 30% had a family history of high cholesterol. Men had higher WC (*p <* 0.01), systolic blood pressure (*p <* 0.01), glucose (*p <* 0.01), creatine (*p <* 0.01), eGFR (*p <* 0.01) than women (Table 2). Interestingly, there were no significant differences between genders in plasma lipids at baseline.

### 3.1. Dietary Records

Dietary cholesterol (746.9 ± 198.7 vs. 110.5 ± 43.0 mg/day) (*p <* 0.001) intake was higher during the egg period mostly due the additional amount of cholesterol provided by eggs, 540 mg (180 mg/egg). In addition, saturated fat, and monounsaturated fat were higher (*p <* 0.001) following EGGS vs CHOLINE (Table 3). Additionally, there was no change observed in polyunsaturated fat and dietary choline. Major micronutrients present in eggs including vitamin E (*p* = 0.026), lutein and zeaxanthin (*p* = 0.018), were higher with intake of 3 eggs per day when comparing to the choline supplement. No change was seen on dietary choline (*p* = 0.745) since intake was matched for both interventions (values were 696.6 ± 97.0 vs. 690.9 vs. 690.9 ± 97.0 mg/day).

### 3.2. Anthropometrics and Lipid Profile

No differences were seen in anthropometric measures such as BMI, systolic BP, and diastolic BP between each intervention (Table 4). Additionally, plasma fasting values of glucose, creatinine, triglycerides, and calculated eGFR were not significantly different among the treatments. Total cholesterol (*p* = 0.040), HDL-C (*p* = 0.030) and LDL-C (*p* = 0.049) were higher after the egg when compared to the choline period. However, no change was observed in the LDL-C/HDL-C ratio.

### 3.3. Apolipoproteins

**Figure 1:**
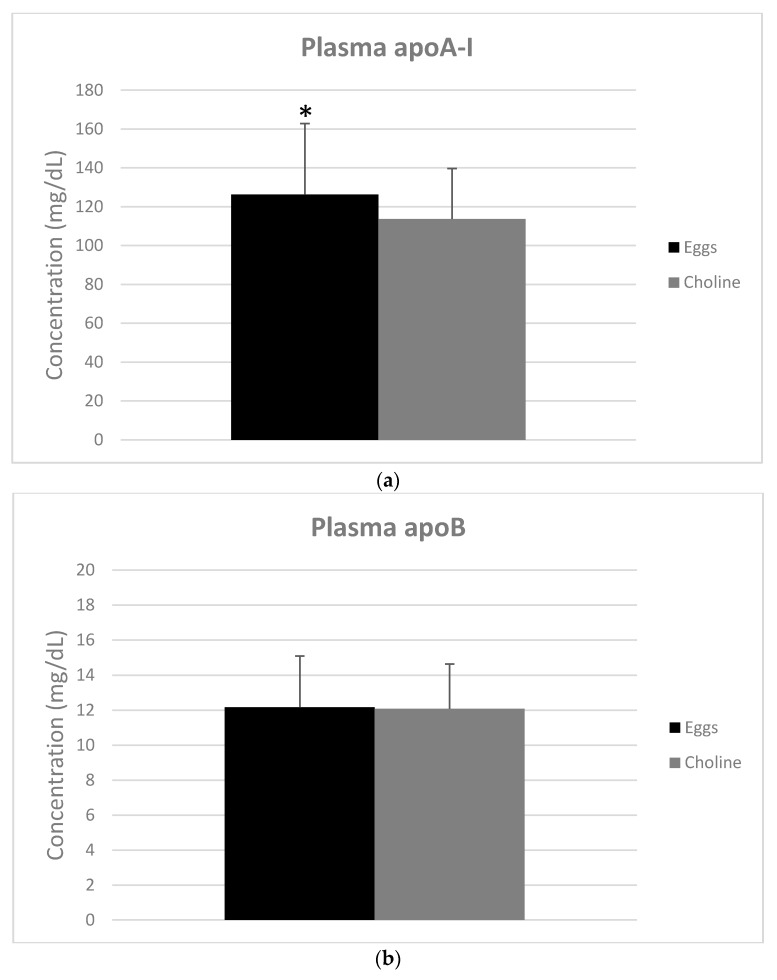

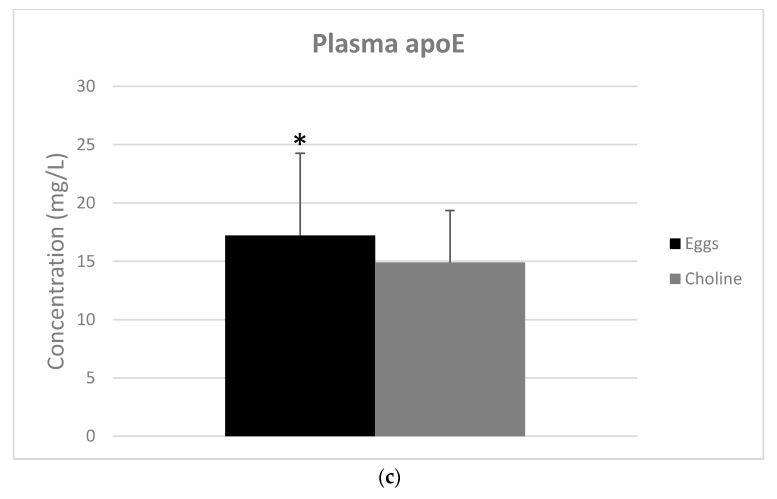
Plasma concentrations of fasting apolipoprotein A-I (**a**); B (**b**); and E (**c**) with intake of 3 eggs versus choline bitartrate supplement for 4 weeks each. Values are presented as mean ± SD for *n* = 29 men and women. Bar with superscripts differ at *p <* 0.05 as determined by paired Student’s *t* test.

Fasting plasma apoA-I (*p* = 0.002) and apoE (*p* = 0.022) concentrations were higher with EGGS vs. CHOLINE (Figure 1a,c). However, there was no difference in the concentration of apoB (Figure 1b).

### 3.4. PBMC Gene Expression

**Figure 2:**
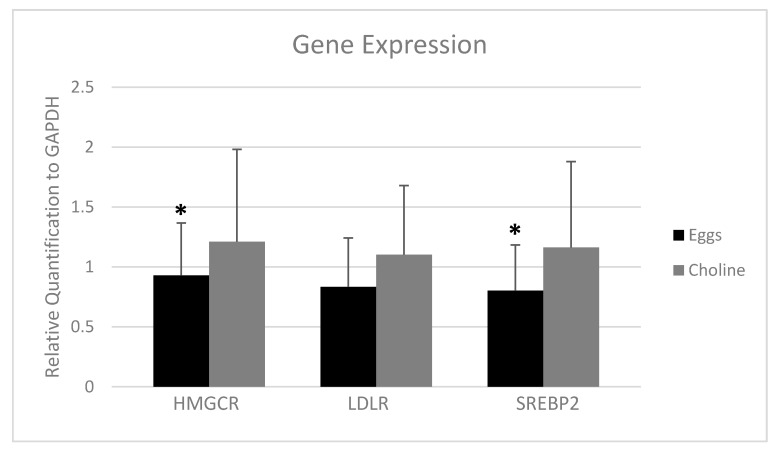
Gene expression of 3-hydroxyl-3-methyl-glutaryl-coenzyme A reductase (HMGCR), low-density lipoprotein receptor (LDLR) and sterol regulatory element-binding protein 2 (SREBP2) with intake of 3 eggs versus choline bitartrate supplement for 4 weeks each. Data were standardized to the expression of GAPDH as a reference gene using the 2^(−ΔΔ*C*t)^ method. Values are presented as mean ± SD for *n* = 27 men and women. Bar with superscripts differ at *p* < 0.05 as determined by paired Student’s *t* test after excluding outliers using Grubb’s test.

In order to investigate whether there were changes in markers of cellular cholesterol biosyntheis/uptake, we measured PBMC gene expression of the rate-limiting biosynthetic enzyme HMGCR, the LDLR, and the key transcription factor involved in their regulation, SREBP2. The expression of HMGCR (*p* = 0.038) and SREBP2 (*p* = 0.008) were lower with EGGS vs CHOLINE (Figure 2). Furthermore, there was a trend (*p* = 0.058) for lower expression of LDLR (Figure 2) with egg intake.

## 4. Discussion

Even with the removal of upper limits for dietary cholesterol from the 2015–2020 DGA [14], egg consumption is still controversial to the majority of the population. The purpose of this study was to evaluate the effects of egg consumption on CVD risk factors and cholesterol metabolism in comparison to a choline bitartrate supplement. In this study, we demonstrated that three eggs per day did not increase the LDL-C/HDL-C ratio compared to choline, a comparator control, and thus does not appear to influence this key risk factor for CVD. Additionally, exogenous cholesterol coming from eggs appears to down regulate the biosynthesis of cholesterol as shown by the lower expression of HMG-CoA reductase and SREBP2 in isolated PBMCs.

Diet is an important modifiable factor that can impact CVD risk and atherosclerosis progression [25]. Mediterranean style diets have many beneficial effects because of the high consumption of fruits, vegetables, and other foods that have low glycemic index [26]. In this aspect, in addition to being low in glycemic index, eggs are also a satiating food which can contribute to low caloric intake [27]. On the other hand, eggs contain 1.6 g saturated fat (SFA) per large egg [12], a concern due to evidence showing diets high in SFA can increase LDL-C, which is a primary factor for CVD [28]. Additionally, the DGA recommends intake of SFA to less than 10% of calories per day [14]. The egg intervention had a higher intake of SFA in comparison to the choline supplementation, which is primarily due to the amount of SFA in three eggs (4.8 g per day) [29]. Participants on the egg intervention were consuming about 35% carbohydrates daily (34.53 ± 8.81) versus 45% with the supplement intake (45.02 ± 11.30). The recommended range for carbohydrate intake is 45–65% of total calories, according to the DGA [14]. Recent observational studies have shown no association between dietary SFA and CVD risk [30,31]. Further research has demonstrated that increased consumption of SFA in the context of a low carbohydrate diet does not significantly increase plasma SFA [32].

Monounsaturated fat (MUFA) is recommended dietary fat to be consumed as the majority of calories coming from fat [33]. Additionally, MUFA are synthesized by the liver after carbohydrate intake [34]. The majority of MUFA in Western diets are oleic acid, which is also one of the fatty acids in the phospholipids present in eggs [13]. The consumption of eggs in comparison to choline supplement had a higher intake of dietary MUFA, and no change in polyunsaturated fat (PUFA). Usually, there’s no specific optimal intake for MUFA, but it is based on the subtraction from the recommended intakes of SFA and PUFA [34]. Some studies have suggested that replacing 5% of energy intake from SFA with MUFA has shown a 15% lower risk for CVD [35]. Therefore, eggs are a great source of healthy fat that can improve diet quality and replace less nutritious foods.

Lutein and zeaxanthin are the major carotenoids present in the egg yolk [36]. One of most important functions of these carotenoids includes the protective effect against age-related macular degeneration that has been prevalently increasing worldwide [37]. With the egg consumption, these dietary carotenoids were higher in comparison to choline bitartrate supplement. Additionally, lutein has shown to have antioxidant activity to lower CVD associated factors such as pro-inflammatory cytokines, aortic and plasma LDL oxidation [38]. Carotenoids are nutraceuticals also known to decrease incidence and prevalence of CVD events [39]. We have demonstrated in other studies that egg consumption increase plasma carotenoids significantly [15,36,40]. Thus, proving the benefits of carotenoids coming from eggs, where they seem to be more bioavailable.

Consuming three eggs per day for four weeks had higher impact on plasma lipids in comparison to choline bitartrate supplements. Total cholesterol, HDL-C and LDL-C concentrations in plasma were higher with the egg intake compared to choline supplementation. In contrast, no change was seen in the LDL-C/HDL-C ratio, which has been shown to be a stronger marker of CVD risk than either LDL-C or HDL-C alone [41]. Importantly, the increase in HDL-C is possibly due to the cholesterol and phospholipids present in egg yolk that are incorporated into HDL. An increase in HDL-C has been previously shown to coincide with increased HDL lipid and antioxidant composition, as well as functional changes in HDL particle bioactivity [15,19,40]. In previous studies, chronic consumption of 1–3 eggs per day was shown to increase plasma HDL-C without elevating other known CVD risk factors in young, healthy populations [15,17,40]. Additionally, apoA-I, the major apolipoprotein associated with HDL, was elevated with egg consumption in the current study. A major function of apoA-I is to facilitate reverse cholesterol transport through the interaction with cholesterol transporters on cells [42]. ApoB-100 is the major structural protein found in very low density lipoprotein (VLDL) and LDL particles [43]. No difference was seen for plasma apoB in this study, while LDL-C increased with EGGS vs CHOLINE. This could be explained by an increase in the cholesterol content of LDL particles rather than the particle number, as there is only one apoB per LDL particle [44]. Of course, higher plasma LDL-C is concerning since LDL can be oxidized/modified and contribute to atherogenesis [6]. Nevertheless, DiMarco et al. observed an increase in LDL particle size with consumption of 3 eggs per day and no change in small LDL, which is known as the most atherogenic lipoprotein [40]. Similarly, increases in both HDL-C and apoA-I with eggs may indicate a greater abundance of HDL particles or larger HDL, the latter has been observed with previous intervention studies involving egg consumption [36,40]. ApoE, present on very-low density lipoproteins and HDL, is important for decreasing plasma cholesterol and clearing triglyceride-rich lipoproteins via the LDLR and LDL-receptor related protein (LRP) [45]. In this context, elevations in apoE with egg intake may complement its effects on HDL to promote RCT to the liver for bile acid synthesis and sterol metabolism [44].

Cholesterol metabolism is regulated at a cellular level and, particularly in the liver, can impact circulating concentrations of plasma cholesterol [2]. A major rate limiting step of cholesterol biosynthesis is controlled by HMGCR, while the LDLR is involved in the cellular uptake of cholesterol from circulating lipoproteins. Consumption of 3 eggs per day showed lower expression of HMGCR and a trend in lowering LDLR. The mechanism proposed here is that dietary cholesterol was able to diminish the hepatic biosynthesis of cholesterol, causing a regulation of extrahepatic cholesterol synthesis as seen with lower expression of HMGCR in PBMCs [46]. SREBP genes are responsible for regulating over 30 genes involved in lipid homeostasis [47]. Specifically, SREBP2 is activated by low intracellular cholesterol levels to promote cholesterol biosynthesis and cholesterol uptake to maintain cellular cholesterol homeostasis. In contrast, increased levels of intracellular cholesterol will downregulate SREBP2 and affect the expression of HMGCR and LDLR [48]. It is possible that we could have observed a significant downregulation of LDLR if the study had been conducted for a longer period of time, but another transcription factor could be regulating the expression of LDLR in this situation [49]. Another limitation of the study is the small number of subjects, considering that there are individual responses to dietary cholesterol; although the small sample size was compensated by the crossover design.

## 5. Conclusions

We have shown in this study that the additional intake of cholesterol from eggs does not increase the risk for heart disease in a young population. Data from PBMC gene expression strongly suggests that cholesterol from eggs downregulates cholesterol biosynthesis and additionally increases HDL cholesterol, leading to the maintenance of the LDL-C/HDL-C ratio.

## Figures and Tables

**Table 1:** Quantitative real-time polymerase chain reaction primer sequences.

Target	Forward Primer	Reverse Primer
HMGCR	5′-CCCAGTTGTGCGTCTTCCA-3′	5′-TTCGAGCCAGGCTTTCACTT-3′
LDLR	5′-ACTGGGTTGACTCCAAACTTCAC-3′	5′-GGTTGCCCCCGTTGACA-3′
SREBP2	5′-GGGGATCCCGATGGACGACAGCGGCGGCT-3′	5′-GGAATTCTCAGTCTGGCTCATCTTTGACCTT-3′
GAPDH	5′-TGTGGGCATCAATGGATTTGG-3′	5′-ACACCATGTATTCCGGGTCAAT-3′

**Table 2:** Baseline characteristic of young, healthy women and men (*n* = 30) participating in 13-week crossover intervention with intake of eggs versus choline supplement for 4 weeks each.

Parameter	Female	Male	*p*-Value
Sex	52%	48%	0.910
Age (years)	25.8 ± 1.95	25.2 ± 2.76	0.679
BMI (kg/m^2^)	23.15 ± 2.46	24.39 ± 6.01	0.498
Waist Circumference (WC)	83.5 ± 4.7	89.8 ± 7.3	0.009
Systolic Blood Pressure (mm Hg)	103.06 ± 9.34	116.00 ± 8.97	0.001
Diastolic Blood Pressure (mm Hg)	68.69 ± 6.17	70.33 ± 7.54	0.387
Glucose (mg/dL)	90.8 ± 4.6	95.5 ± 3.6	0.003
Creatinine (mg/dL)	0.79 ± 0.10	0.98 ± 0.10	0.001
eGFR (mL/min)	94.98 ± 14.65	99.39 ± 10.94	0.001
Triglycerides (mg/dL)	64.31 ± 27.09	70.31 ± 41.00	0.640
Total Cholesterol (mg/dL)	166.56 ± 30.66	159.92 ± 29.62	0.561
HDL-C (mg/dL)	70.81 ± 11.93	68.46 ± 7.73	0.545
LDL-C (mg/dL)	82.89 ± 26.51	77.40 ± 25.33	0.576
LDL-C/HDL-C	1.20 ± 0.39	1.16 ± 0.45	0.814

Values are presented as mean ± SD.

**Table 3:** Dietary records for fats and carotenoids of healthy, young population (*n* = 29) at the end of each intervention arm, eggs versus choline supplement intake for 4 weeks each.

Nutrient	EGGS	CHOLINE	*p*-Value
Saturated Fat (%)	13.44 ± 4.46	10.91 ± 3.58	<0.001
Monounsaturated Fat (g)	29.44 ± 8.84	22.42 ± 7.68	<0.001
Polyunsaturated Fat (g)	16.09 ± 6.48	15.72 ± 5.97	0.809
Lutein + Zeaxanthin (μg)	1474.34 ± 724.72	1115.41 ± 746.15	0.018

Values are presented as mean ± SD.

**Table 4:** Anthropometrics measures and fasting plasma biochemical parameters of subjects (*n* = 29) at the end of each intervention arm, three eggs versus choline bitartrate supplement intake for 4 weeks each.

Parameter	Eggs	Choline	*p*-Value
BMI (kg/m^2^)	24.1 ± 2.8	24.0 ± 2.60	0.347
Systolic Blood Pressure (mm Hg)	108.1 ± 10.7	108.9 ± 10.9	0.604
Diastolic Blood Pressure (mm Hg)	68.8 ± 7.70	68.8 ± 6.3	0.939
Glucose (mg/dL)	92.3 ± 6.0	90.9 ± 5.7	0.226
Creatinine (mg/dL)	0.85 ± 0.11	0.86 ± 0.13	0.415
eGFR (mL/min)	100.6 ± 12.3	99.5 ± 12.9	0.553
Triglycerides (mg/dL)	69.6 ± 29.5	73.6 ± 36.0	0.355
Total Cholesterol (mg/dL)	172.6 ± 35.8	162.7 ± 30.7	0.040
HDL-C (mg/dL)	61.0 ± 16.0	57.0 ± 14.3	0.030
LDL-C (mg/dL)	97.7 ± 31.7	90.9 ± 26.3	0.049
LDL-C/HDL-C	1.72 ± 0.72	1.70 ± 0.67	0.775

Values are presented as mean ± SD. Student’s *t* test was used to determine statistical significance.
